# Cardiovascular dysautonomia in Achalasia Patients: Blood pressure and heart rate variability alterations

**DOI:** 10.1371/journal.pone.0248106

**Published:** 2021-03-15

**Authors:** Ana Leonor Rivera, Bruno Estañol, Julio J. Macias-Gallardo, Guillermo Delgado-Garcia, Ruben Fossion, Alejandro Frank, Gonzalo M. Torres-Villalobos

**Affiliations:** 1 Instituto de Ciencias Nucleares, Universidad Nacional Autónoma de México, Ciudad Universitaria, Coyoacan, Mexico City, Mexico; 2 Centro de Ciencias de la Complejidad, Universidad Nacional Autónoma de México, Ciudad Universitaria, Coyoacan, Mexico City, Mexico; 3 Department of Neurology and Psychiatry and Clinical Neurophysiology Laboratory, Instituto Nacional de Ciencias Médicas y Nutrición Salvador Zubirán, Tlalpan, Mexico City, Mexico; 4 Department of Clinical Neurosciences, University of Calgary, Calgary Alberta, Canada; 5 El Colegio Nacional, Centro Histórico, Mexico City, Mexico; 6 Department of Experimental Surgery, Instituto Nacional de Ciencias Médicas y Nutrición Salvador Zubirán, Tlalpan, Mexico City, Mexico; TNO, NETHERLANDS

## Abstract

Achalasia is a disease characterized by the inability to relax the esophageal sphincter due to a degeneration of the parasympathetic ganglion cells located in the wall of the thoracic esophagus. Achalasia has been associated with extraesophageal dysmotility, suggesting alterations of the autonomic nervous system (ANS) that extend beyond the esophagus. The purpose of the present contribution is to investigate whether achalasia may be interpreted as the esophageal manifestation of a more generalized disturbance of the ANS which includes alterations of heart rate and/or blood pressure. Therefore simultaneous non-invasive records of the heart inter-beat intervals (IBI) and beat-to-beat systolic blood pressure (SBP) of 14 patients (9 female, 5 male) with achalasia were compared with the records of 34 rigorously screened healthy control subjects (17 female, 17 male) in three different conditions: supine, standing up, and controlled breathing at 0.1 Hz, using a variety of measures in the time and spectral domains. Significant differences in heart rate variability (HRV) and blood pressure variability (BPV) were observed which seem to be due to cardiovagal damage to the heart, i.e., a failure of the ANS, as expected according to our hypothesis. This non-invasive methodology can be employed as an auxiliary clinical protocol to study etiology and evolution of achalasia, and other pathologies that damage ANS.

## Introduction

Achalasia, a cause of esophageal motor disorders, is an autoimmune disease with a failure of the lower esophageal sphincter to relax after swallowing [1]. It is characterized by degeneration of parasympathetic ganglion cells in its myenteric plexus [2, 3]. Clinical manifestations include dysphagia, regurgitation, chest pain, cough, aspiration, weight loss and heartburn [4, 5]. It affects both sexes and all ages with an annual incidence of 0.3–1.6/100,000 [6]. The diagnosis is confirmed by high resolution manometric studies (HRM) [7]. In a Mexican population no difference in bio-geographic ancestry between patients and controls occur, however, the frequency of some alleles and extended haplotypes are increased [8].

Achalasia has a widespread effect on the body. Extraesophageal denervation has been found in the stomach [9], the vagal trunk and the dorsal motor nuclei [10–16]. Functional and motor abnormalities have been observed in the stomach [17–22], the gallbladder [19], and the small bowel [23, 24]. It has an important local and systemic inflammatory autoimmune component, associated with the presence of specific anti-myenteric autoantibodies, as Herpes Simplex Virus (HSV-1) infection [2]. Moreover, achalasia may occur in primary autonomic dysfunction diseases, such as orthostatic hypotension [25], acute autonomic neuropathy [26], among others [27–30], and in secondary autonomic disorders, such as diabetes mellitus [31–36]. Autonomic reflex tests (Ewing battery) and statistical measures of heart rate variability (HRV) on achalasia subjects are inconclusive, as half of the studies detected autonomic alterations [37–40], but the other half did not [41–44].

Autonomous nervous system (ANS) regulates blood pressure homeostasis [45] by the cardiac cycle through central (e.g., vasomotor and respiratory centers) and peripheral (e.g., arterial pressure and respiratory movements) oscillators. Parasympathetic modulation decreases the heart rate and cardiac contractility, whereas activity of the sympathetic branch opposes these effects and regulates peripheral vasoconstriction [46–49]. Thus, ANS activity can be evaluated through HRV [47, 48, 50–56], and by the homeostatic regulatory mechanism [45, 57–59]. This strategy allowed us to increase the statistical significance to distinguish between controls, recently diagnosed and long-standing diabetic patients [54, 55]. Short term HRV has a temporal structure with robust long-range correlations, with fractal and non-linear features that break down under pathologic conditions like Diabetes Mellitus (DM), reflecting changes in the neuro-autonomic control mechanisms [54, 55, 60–66]. HRV has been used as an early sign of DM autonomic neuropathy [54, 55], but has not been studied for achalasia.

Systolic blood pressure (SBP) variability in control subjects changes under different maneuvers [49], and during active standing and handgrip reflecting lower parasympathetic cardiac activity at rest [67]. Interestingly, whereas HRV at rest appears to be a protective health factor [45], it has been suggested that SBP variability is a risk factor [67–70]. Therefore, it would be interesting to see how blood pressure changes in patients with achalasia. To our knowledge this type of study has not been previously performed.

The objective of the present contribution is to evaluate extraesophageal autonomic function in achalasia. To do that, we compare blood pressure and heart rate variability between controls and patients with achalasia in 3 selected conditions: clinostatism, orthostatism, and rhythmic breathing at 0.1Hz. As far as we know, this is the first analysis involving simultaneous blood pressure and heart rate variability data using quantitative measures in the time and frequency domains to compare control subjects and patients with achalasia. Our hypothesis is that achalasia may be associated to damage to the parasympathetic fibers to the sinus node and some other areas of the heart innervated by the vagal cardiovascular innervation. If the parasympathetic neurons of the myenteric plexus of the esophagus are damaged there is a strong possibility that the cholinergic neurons that innervate the sinus auricular node are also damaged and therefore this denervation could be demonstrated analyzing the blood pressure and heart rate variability.

## Research design and methodology

All subjects provided written informed consent, their medical history was screened and underwent a physical examination. The Ethical Committee of the Instituto Nacional de Ciencias Médicas y Nutrición Salvador Zubirán approved the protocol for the physiological monitoring of the achalasia patients.

SBP and HR data were recorded simultaneously with a Portapress^®^ device of Finapres Medical Systems, The Netherlands [71]. The Portapress^®^ quantifies the blood pressure waveform at the finger with a precision of 1 mmHg and a time resolution of 1 ms, which allows us to derive other hemodynamic parameters. Here only the IBI and the SBP are analyzed, because the diastolic blood pressure (DBP) has an analogous time series as SBP as seen in S1 Fig. IBI is measured in units of seconds and SBP as mmHg. The detailed methodology for time series analysis is given in the S1 Appendix.

Student’s *t* test was used to compare the different study groups of control subjects and achalasia patients. A value of *p*<0.05 was considered statistically significant in the hypothesis test of different means of the moments (standard deviation, skewness and kurtosis), HMP α, LF/HF, frequency radius r_f_, and resonance parameter β.

## Results and discussion

The study population was composed of 48 subjects clinically classified in 2 distinct groups as follows:

- 34 healthy control subjects, 17 women with a body mass index (BMI = height/weight^2) from 19.1 to 28.5 kg/m^2^ (with a mean and standard deviation of (24±3) kg/m^2^), aged from 21 to 50 years old, (35±8) years old, and 17 men with BMI from 19.5 to 28.7 kg/m^2^, (25±3) kg/m^2^, aged from 20 to 50 years old, (30±7) yr. Subjects were classified as controls if they did not smoke, had no cardiac diseases, and did not take medication. They were not hypertensive and had blood pressure levels below 120/80 mmHg.
- 14 patients with achalasia, 9 women with BMI from 19.6 to 35.7 kg/m^2^, (25±4) kg/m^2^, aged between 25 and 70 years old, (49±15) yr; and 5 men with BMI from 21.0 to 35.8 kg/m^2^, (28±5) kg/m^2^, aged between 30 and 63 years old, (41±13) yr. These patients were diagnosed with achalasia by a team of gastroenterologists.

Achalasia patients were all asymptomatic of cardiovascular disease. They did not have syncope or presyncope. Neither they had palpitations, arrhythmias or symptoms or signs of heart failure. They were not hypertensive. Control subjects and achalasia patients abstained from caffeine, beta-blockers, anticholinergics, antihistamines, opioids and adrenergic medication for the 48 hours before the test. Data consisted of short-term 5 minutes recordings measured non-invasively with the Portapress^®^ equipment. IBI and SBP were registered simultaneously while the subject was in supine position. Then, subjects were made to stand up, relaxed for 1 minute and stayed in this position for another 5 minutes. Finally, a controlled breathing test at 0.1 Hz was realized (subjects were asked to inspire and expire at six breaths per minute, 5 seconds in and 5 seconds out when standing up).

**Fig 1:**
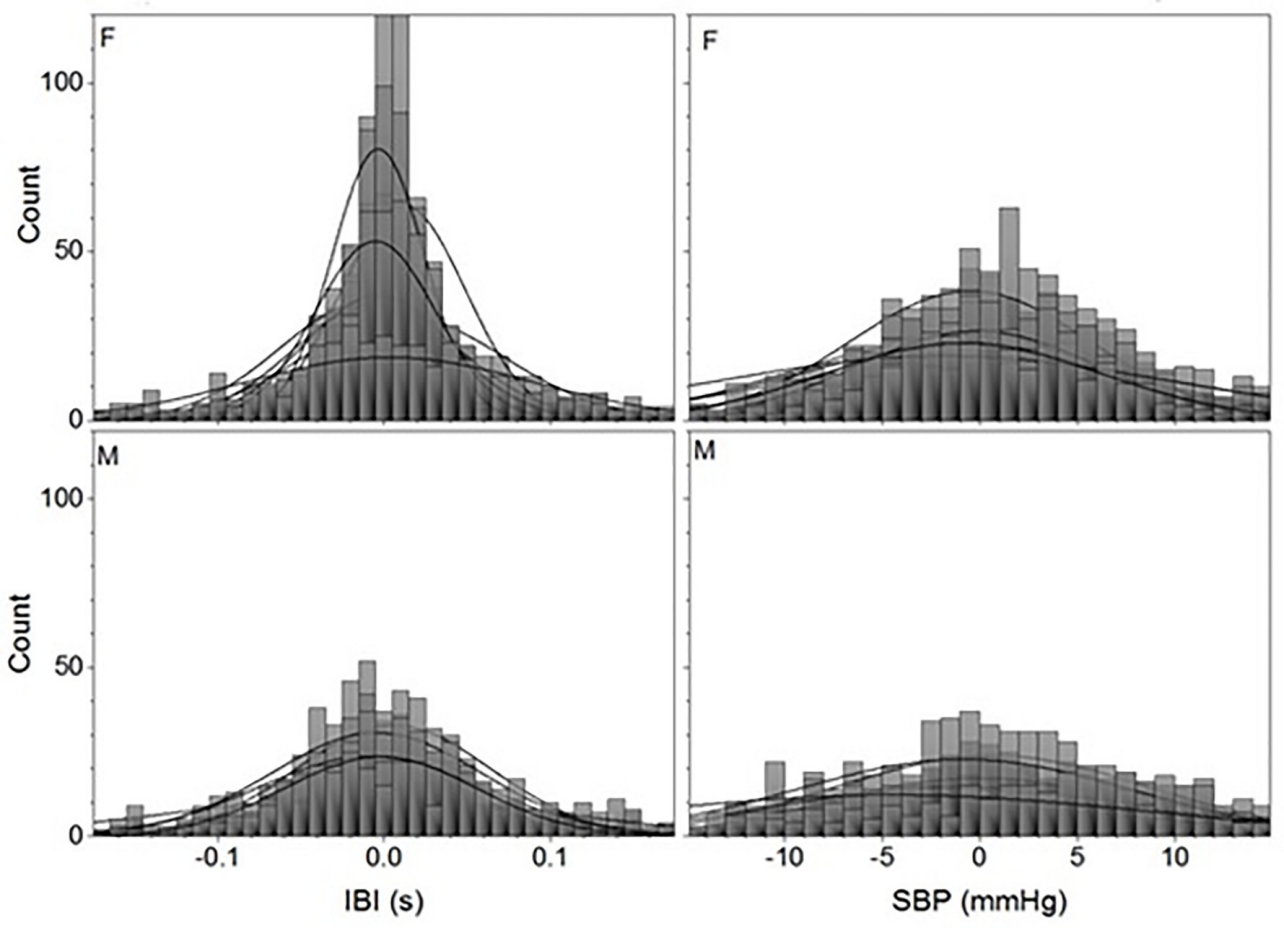
Histograms of detrended time series for achalasia patients standing up. IBI records (left-hand panels) and SBP (right-hand panels) for all 9 female (F) (upper row) and all 5 male (M) (bottom row) patients are shown with the best normal distribution fit (continuous lines).

Histograms of the achalasia patients show a clear gender difference. IBI and SBP for all the included autonomic reflex tests have more rigid, symmetric, and mesokurtic histograms for male patients than for female achalasia patients, as seen in Fig 1 which shows the overlapping histograms of all the patients standing up.

Even when the histograms for each subject have tails and do not follow Gaussian distributions, the moments for each group of study (30 control subjects, 14 achalasia patients) follow Normal distributions (verified by different normality test: Shapiro-Wilk, Kolmogorov-Smirnov, Anderson-Darling, and Chen-Shapiro).

**Fig 2:**
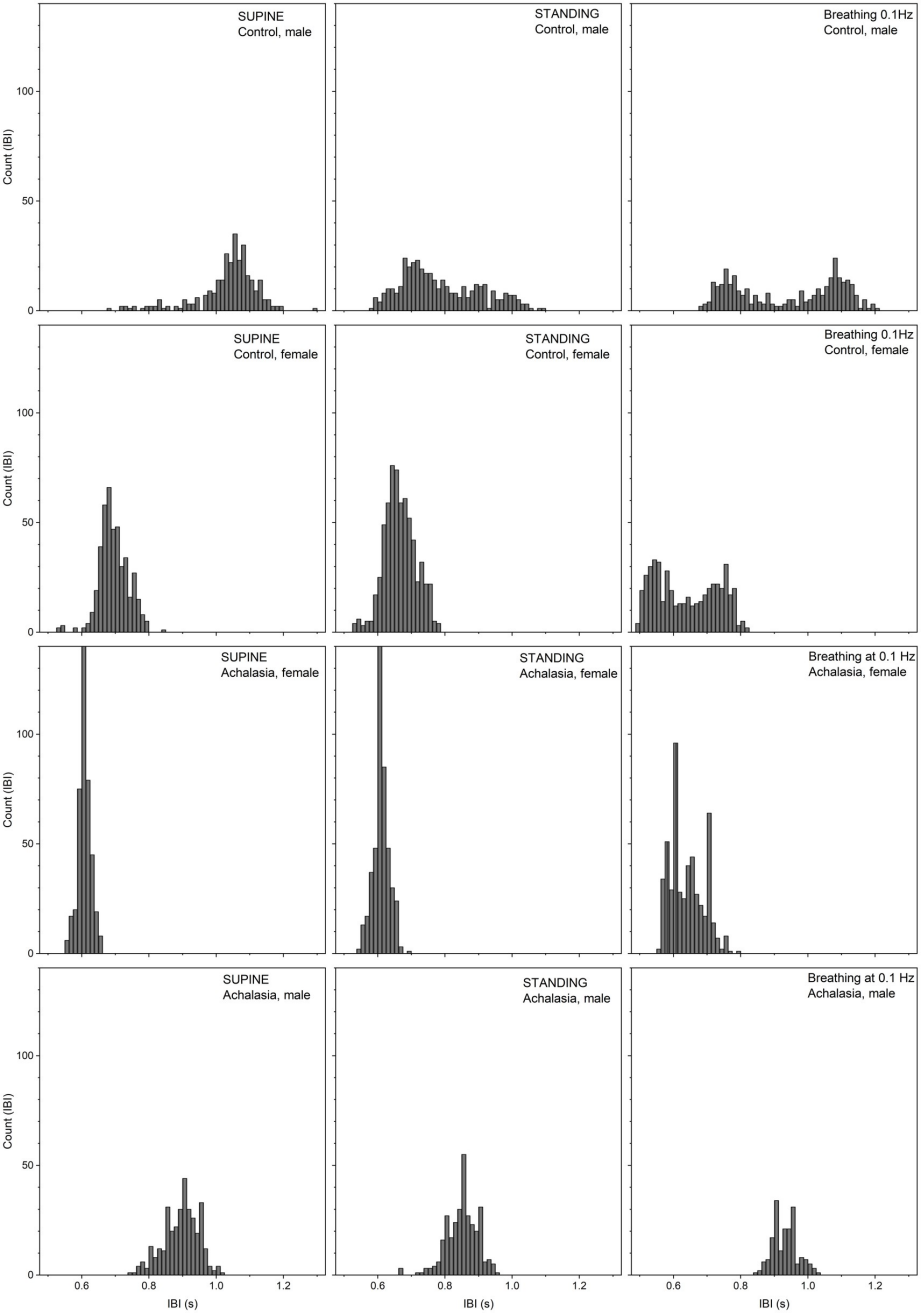
Histograms of IBI detrended time series for typical patients (30 years old). Male M30C01 and female F30C01 control (upper rows), female F30A01 achalasia (middle row), and male M30A01 achalasia patients (lower row) in supine position (left-hand column), standing up position (middle column), and under controlled breathing at 0.1 Hz (right-hand column).

**Fig 3:**
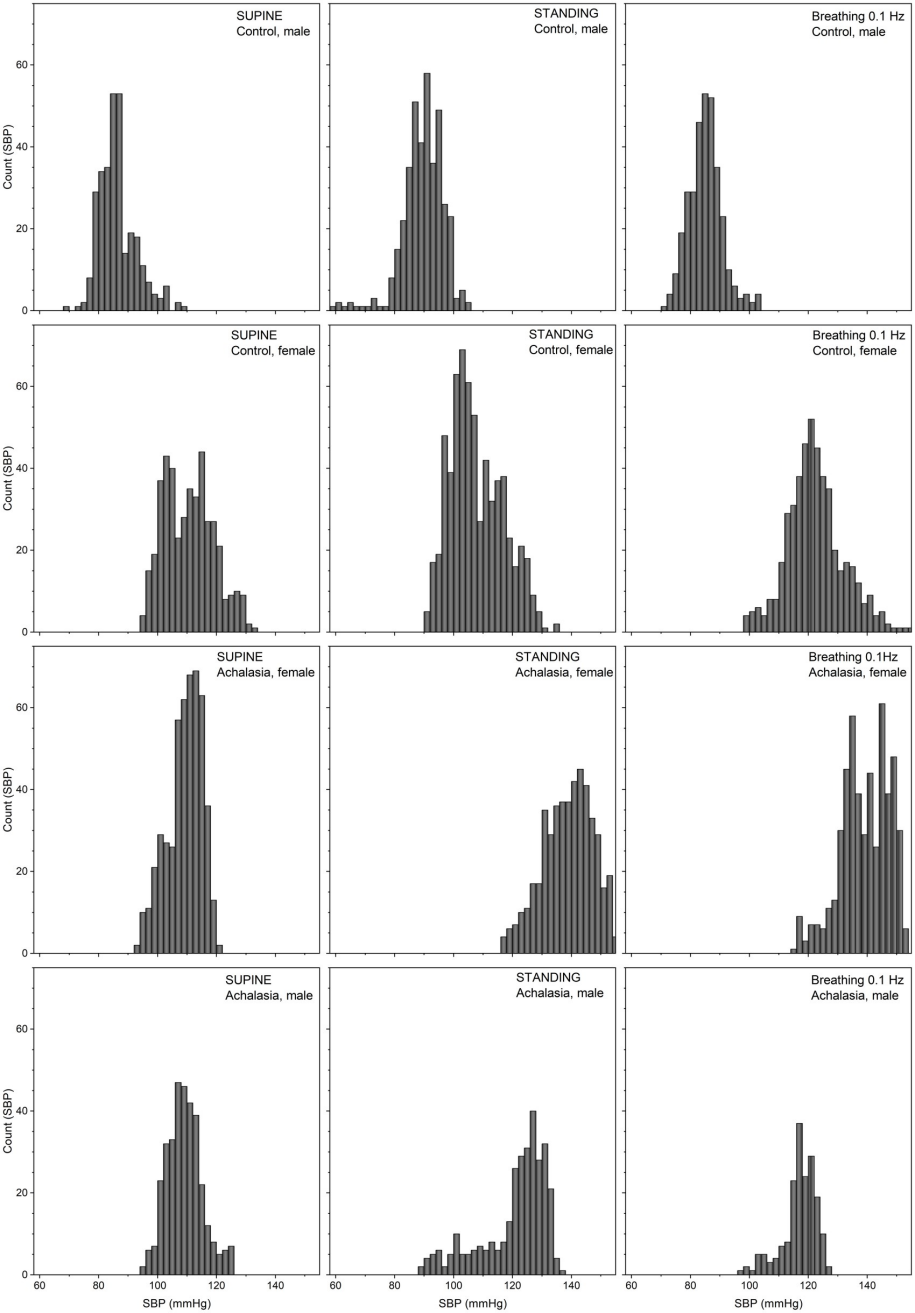
Histograms of SBP detrended time series for typical patients (30 years old). Male M30C01 and female F30C01 control (upper rows), female F30A01 achalasia patients (middle row) and male M30A01 achalasia patients (lower row) on supine position (left-hand column), standing up (middle column), and under controlled breathing at 0.1 Hz (right-hand column).

Results for the statistical moments of IBI records are in Table 1, while those of SBP are in Table 2. Results for control subject values in Tables 1 and 2 were calculated as a reference [72]. Average and standard deviations of our controls agree with previous publications [73]. Histograms of the detrended IBI records for a typical female and male control, female (F) and male (M) achalasia patients, all with similar BMI and age, for supine position, standing up position, and under controlled breathing at 0.1 Hz test are plotted in Fig 2, while Fig 3 shows the SBP. Continuous curves correspond to the best normal distribution fit to the histograms. Histograms of IBI and SBP signals during controlled breathing tend to be more platykurtic than the corresponding ones for supine and standing up positions. From these figures, it is evident that blood pressure shows less variability than heart rate. HRV is more altered during the standing position. SBP histograms are more disperse and platykurtic than IBI ones, in fact, standing up, the SBP distribution is flatter over a wider range. Moreover, for male achalasia patients, the IBI record does not show a strong difference under supine, standing up, or controlled breathing (Table 1) indicating a loss of adaptation capacity (less HRV) to different stressors, in contrast to control female subjects that have statistically significant changes under positional changes [49].

**Table 1:** Heart interbeat interval in the time domain.

	Maneuver	Sex	μ (s)	SD (s)	*sk*	κ	SD/μ
**Controls**	Supine	F	0.9 ± 0.1	0.05 ± 0.02	-0.2 ± 0.5	1 ± 1	0.05 ± 0.02
		M	1.0 ± 0.1	0.06 ± 0.02	-0.3 ± 0.4	0.4 ± 0.8	0.06 ± 0.02
	Standing up	F	0.8 ± 0.1	0.05 ± 0.02	0.1 ± 0.4	0.03 ± 0.6	0.06 ± 0.03
		M	0.8 ± 0.1	0.07 ± 0.04	0.3 ± 0.5	0.2 ± 1	0.09 ± 0.03
	Breathing at 0.1 Hz	F	0.8 ± 0.1	0.09 ± 0.03	0.0 ± 0.4	-0.8 ± 0.8	0.11 ± 0.04
		M	0.9 ± 0.1	0.09 ± 0.04	0.1 ± 0.4	-0.6 ± 0.8	0.10 ± 0.04
**Achalasia**	Supine	F	0.8 ± 0.1	0.03 ± 0.01* (*p* = 0.007)	0.2 ± 0.6* (*p* = 0.01)	0.7 ± 2	0.04 ± 0.01
		M	1.0 ± 0.2	0.05 ± 0.02	-0.4 ± 0.1	0.4 ± 1	0.06 ± 0.03
	Standing up	F	0.7 ± 0.1	0.05 ± 0.02	-0.8 ± 1* (*p* = 0.04)	5 ± 6* (*p* = 0.03)	0.06 ± 0.02
		M	0.9 ± 0.2	0.08 ± 0.02	-0.4 ± 0.4* (*p* = 0.04)	0.7 ± 0.7* (*p* = 0.04)	0.08 ± 0.02
	Breathing at 0.1 Hz	F	0.8 ± 0.1	0.06 ± 0.03* (*p* = 0.002)	0.1 ± 0.4	-0.6 ± 0.8	0.08 ± 0.04
		M	0.9 ± 0.1	0.08 ± 0.03	0.2 ± 0.1	-1.0 ± 0.5	0.10 ± 0.03

* indicates statistically significant difference with respect to control (*p* value <0.05).

**Table 2:** Systolic blood pressure in the time domain.

	Maneuver	Sex	μ (mmHg)	SD (mmHg)	*sk*	κ	SD/μ
**Controls**	Supine	F	100 ± 10	5 ± 2	0.6 ± 0.6	0.9 ± 3	0.05 ± 0.02
		M	110 ± 10	6 ± 3	0.3 ± 0.5	0.2 ± 1	0.05 ± 0.02
	Standing up	F	110 ± 10	6 ± 3	-0.02 ± 0.7	0.6 ± 2	0.06 ± 0.03
		M	100 ± 10	6 ± 2	-0.03 ± 0.5	0.3 ± 0.9	0.06 ± 0.02
	Breathing 0.1 Hz	F	110 ± 20	8 ± 3	0.07 ± 0.3	-0.3 ± 0.4	0.07 ± 0.02
		M	110 ± 20	7 ± 2	-0.04 ± 0.5	0.08 ± 1	0.06 ± 0.02
**Achalasia**	Supine	F	100 ± 20	5 ± 2	0.2 ± 0.4	-0.1 ± 0.5* (*p* = 0.03)	0.05 ± 0.02
		M	100 ± 10	6 ± 1	0.1 ± 0.5	-0.4 ± 0.4* (*p* = 0.04)	0.06 ± 0.01
	Standing up	F	110 ± 20	9 ± 2* (*p* = 0.4)	-1.1 ± 0.8* (*p* = 0.02)	4 ± 4* (*p* = 0.02)	0.07 ± 0.01
		M	110 ± 10	10 ± 2* (*p* = 0.3)	-0.5 ± 0.3* (*p* = 0.04)	0.4 ± 1	0.09 ± 0.02 * (*p* = 0.04)
	Breathing 0.1 Hz	F	110 ± 20	7 ± 2	0.04 ± 0.5	-0.09 ± 1* (*p* = 0.03)	0.07 ± 0.02
		M	104 ± 9	8 ± 2	-0.3 ± 0.6* (*p* = 0.01)	0.1 ± 0.9* (*p* = 0.04)	0.08 ± 0.02 * (*p* = 0.04)

* indicates statistically significant difference with respect to control (*p* value <0.05).

The HRV of achalasia patients have in supine position, IBI Gaussian distributions (null sk and κ) with small SD/μ (rigid behavior); while standing up, IBI distributions have tails to the left (negative sk), are leptokurtic (κ positive) with larger SD/μ and range than in supine position (SD is almost the double); while IBI rhythmic breathing distributions are symmetric (null sk), platykurtic (κ negative) with similar values of SD/μ and range than standing up (see Fig 2, Table 1). Comparing with control, there is a statistically significant difference on IBI in supine and standing up positions for female achalasia patients while male only differ standing up (Table 1), reflecting more rigid distributions for achalasia female patients.

With respect to the SBPV of achalasia patients, SBP supine distributions are almost Gaussian (null sk and κ) with small SD/μ (rigid behavior); standing up, SBP distributions have tails to the left (negative sk), are leptokurtic (κ positive) with larger SD/μ and range than in supine position (SD is the double); while SBP rhythmic breathing distributions are almost Gaussian (null sk and κ) with similar values of SD/μ than standing up (see Fig 3, Table 2). Comparing with control, there is a statistically significant difference on SBP symmetry for achalasia patients on all maneuvers, in standing up maneuvers for female achalasia patients while male differ standing up and during rhythmic breathing (Table 2).

**Fig 4:**
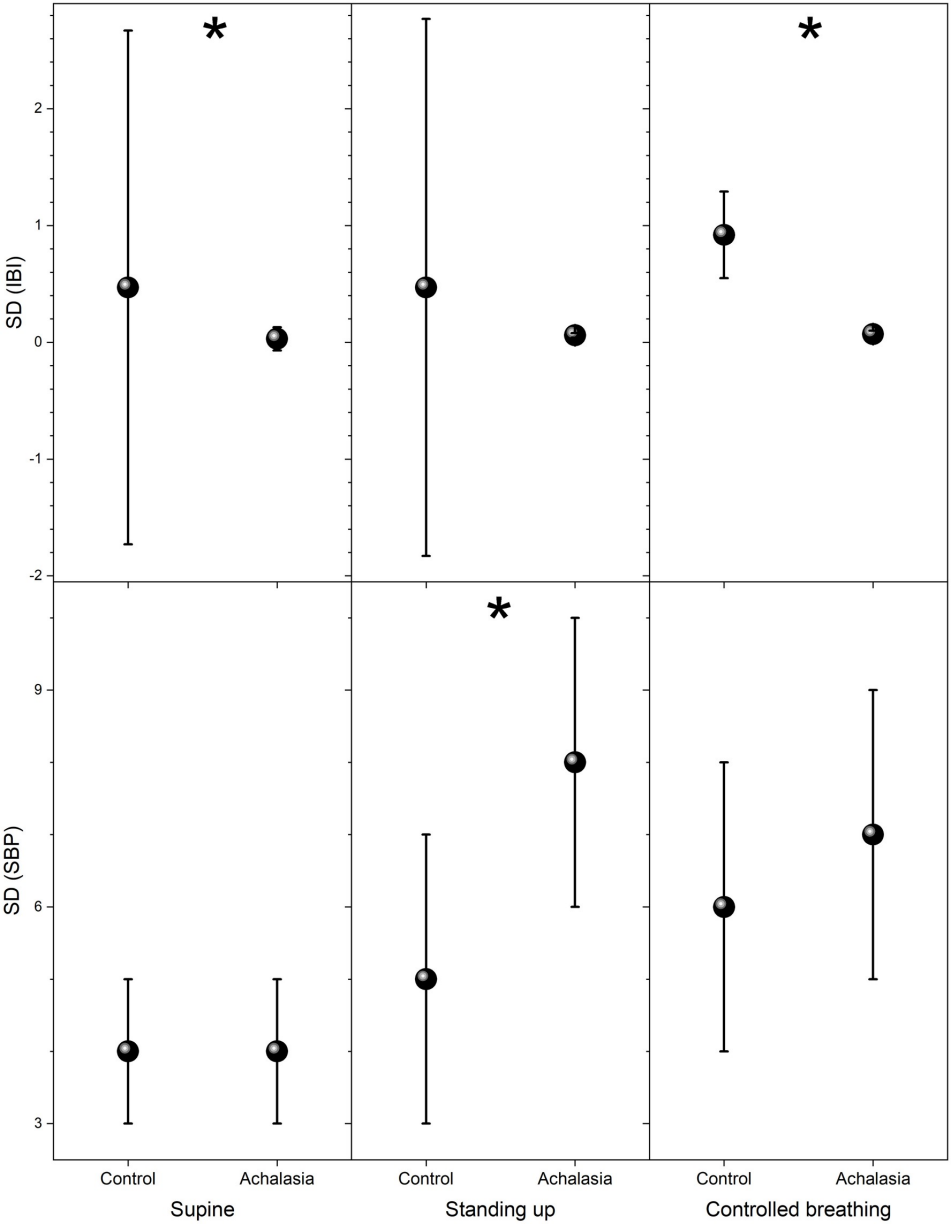
Standard deviation of detrended data for the different groups for IBI (upper panels) and SBP (bottom panels) during supine position (left-hand panels), standing up (middle panels), and controlled breathing (right-hand panels). The vertical crosshair corresponds to one standard deviation around the population average, while the dot is the median; * indicates statistically significant difference with respect to control (*p* value < 0.05).

**Fig 5:**
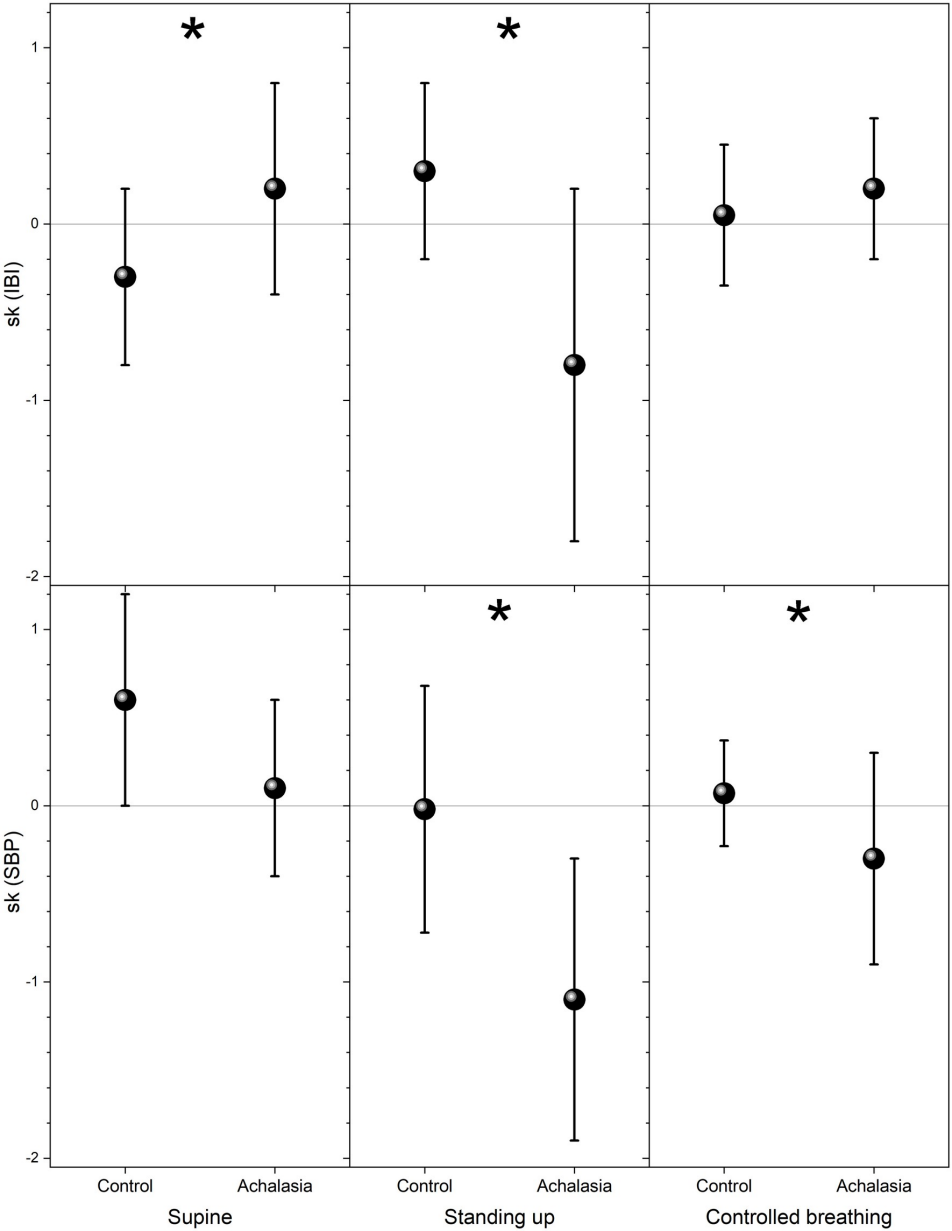
Skewness with respect to the median for control and achalasia groups during supine position (left-hand panels), standing up (middle panels), and controlled breathing (right-hand panels), IBI (top row), and SBP (bottom row). The vertical crosshair corresponds to one standard deviation around the population average, while the dot is the median; * indicates statistically significant difference with respect to control (*p* value <0.05).

**Fig 6:**
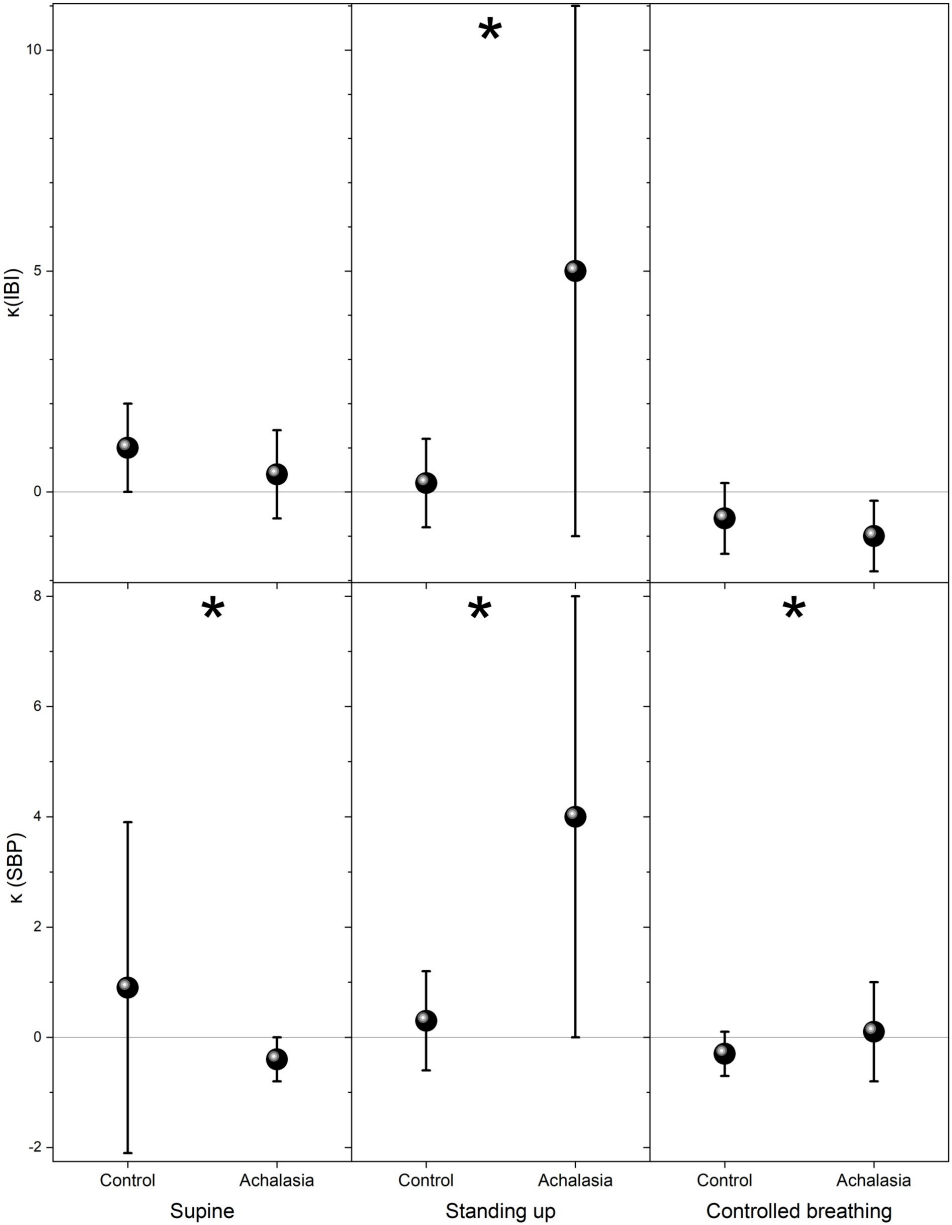
Kurtosis with respect to the median for control and achalasia groups during supine position (left-hand panels), standing up (middle panels), and controlled breathing (right-hand panels), IBI (top row) and SBP (bottom row). The vertical crosshair corresponds to one standard deviation around the population average, while the dot is the median; * indicates statistically significant difference with respect to control (*p* value <0.05).

The usual parameter employed in HRV analysis to distinguish health from illness is the standard deviation of the IBI and SBP records, which we plot for comparison in Fig 4. For IBI, statistically significant separation of the groups is on the controlled breathing test, while for SBP is standing up (see Tables 1 and 2). Higher statistical moments of the distribution are shown in Fig 5 (skewness with respect to the median) and in Fig 6 (kurtosis with respect to the median) for control subjects and achalasia patients’ groups during all the autonomic reflex tests. Qualitatively, a negative skewness indicates that the tail on the left side of the probability density function is longer than the right side and the bulk of the values lie to the right of the mean, while a positive skewness indicates that the bulk of the values lie to the left of the mean. In these graphs, it is possible to visually separate the different groups, especially for IBI in supine position and SBP under controlled breathing. In all these tests, control subjects present a larger dispersion of the data in IBI and SBP records than achalasia patients (Tables 1 and 2).

Table 3 resumes the results for IBI analysis in the frequency domain for control [72] and achalasia patients. For achalasia patients, HF is the same for all the maneuvers, LF and r_F_ standing up or in supine position are similar, while under rhythmic breathing, LF and r_F_ are one order of magnitude larger, LF/HF increases from supine position to standing up and doubles under controlled breathing. Power spectral density (PSD) standing up is almost scale invariant (slope close to -1). For rhythmic breathing at 0.1 Hz, the PSD shows stronger time series correlation, more like Brownian motion than free scale. Under controlled breathing at 0.1 Hz, achalasia patients also show a characteristic resonance peak showing that they do not lose the cardio-respiratory coupling, in contrast with diabetic patients [55]. However, LF, HF and r_F_ are statistically significant different for all maneuvers for achalasia patients (Table 3).

**Table 3:** Interbeat interval in the frequency domain.

	Maneuver	Sex	LF (s^2^) (0.04–0.15 Hz)	HF (s^2^) (0.14–0.4 Hz)	LF/HF	r_F_ (s^2^) $\sqrt{{\text{LF}}^{2}+{\text{HF}}^{2}}$	slope PSD (s^2^)	β
**Control**	Supine	F	6 ± 3	9 ± 3	0.7 ± 0.2	11 ± 4	-0.6 ± 0.5	
		M	7 ± 3	10 ± 4	0.7 ± 0.3	12 ± 4	-1.0 ± 0.4	
	Standing up	F	10 ± 3	11 ± 4	0.9 ± 0.3	15 ± 4	-0.8 ± 0.6	
		M	12 ± 5	11 ± 3	1.2 ± 0.3	16 ± 6	-1.1 ± 0.9	
	Breathing at 0.1 Hz	F	16 ± 7	12 ± 6	1.5 ± 0.4	20 ± 9	-1.2 ± 0.4	0.29 ± 0.07
		M	15 ± 5	10 ± 4	1.5 ± 0.4	18 ± 6	-1.2 ± 0.3	0.3 ± 0.1
**Achalasia**	Supine	F	4 ± 2	5 ± 3* (*p* = 0.01)	0.8 ± 0.2	6 ± 4* (*p* = 0.002)	-0.3 ± 0.9	
		M	5 ± 1*	7 ± 1* (*p* = 0.02)	0.7 ± 0.2	9 ± 2* (*p* = 0.007)	-0.4 ± 0.8 * (*p* = 0.01)	
	Standing up	F	4 ± 2*	4 ± 2*(*p* = 0.0005)	1.0 ± 0.2	6 ± 3* (*p* = 0.0001)	-0.5 ± 0.7	
		M	8 ± 3*	6 ± 1* (*p* = 0.003)	1.4 ± 0.4	10 ± 3* (*p* = 0.01)	-0.8 ± 0.6 * (*p* = 0.02)	
	Breathing at 0.1 Hz	F	14 ± 6	6 ± 1* (*p* = 0.03)	2.5 ± 0.9* (*p* = 0.02)	15 ± 6	-1.7 ± 0.7 * (*p* = 0.02)	0.3 ± 0.1
		M	18 ± 4	6 ± 2* (*p* = 0.04)	3.1 ± 0.7* (*p* = 0.01)	19 ± 4	-1.7 ± 0.7 * (*p* = 0.02)	0.3 ± 0.2

* indicates statistically significant difference with respect to control (*p* value <0.05).

Results for the homeostatic measure parameter α appear in Table 4. For achalasia patients, HMP α is similar under all maneuvers, reflecting that for the achalasia patients it is more difficult to respond to external changes, they have parameters that are more rigid but during the upright position also the ratio between the frequency radius of IBI and SBP decreases significantly. Controlled breathing allows us to distinguish the populations. During this test, control subjects have higher values of α with large SD reflecting the correlation between IBI and SBP. With achalasia, HMPα diminishes not only in value but also in variability. That is, not only the heart response becomes more rigid, but the variability of the blood pressure also increases, in agreement with HRV being a protective health factor and blood pressure variability a risk factor.

**Table 4:** Homeostatic measure parameter α.

Maneuver	Sex	α for control	α for achalasia
Supine	F	2.1 ± 2	1.9 ± 2
	M	2.6 ± 3	1.5 ± 1 * (*p* = 0.03)
Standing up	F	1.1 ± 0.9	2.4 ± 3 * (*p* = 0.04)
	M	1.3 ± 1	1.4 ± 1
Rhythmic breathing (0.1 Hz)	F	2.2 ± 1	2.1 ± 1
	M	1.5 ± 1	1.7 ± 1

* indicates statistically significant difference with respect to control (*p value* <0.05).

## Conclusions

The analysis of IBI and SBP detrended time series in the time domain shows that all moments of the distribution are relevant parameters that allow significant differentiation between control subjects and achalasia patients, they also indicate harm to the parasympathetic damage to the heart that is concomitant to the vagal damage to the esophagus. The analysis in this study is relatively simple to perform in patients, since it only employs the moments of the distributions and their ratios, so it can be relatively easily adapted to clinical inspection and quantitative evaluation.

## Supporting information

S1 Fig SBP and DBP of a control man 21 years old breathing rhythmically at 0.1 recorded by the Portapress^®^. (PDF)

S2 Fig Trend removal using empirical mode decomposition. (PDF)

S3 Fig Equidistant time-series of the interbeat interval (IBI) generated by cubic-spline interpolation. Dots are detrended data points and continuous line is interpolation.
(PDF)

S1 Appendix Analysis methodology [74–79]. (DOCX)
