# Supplementary material for: Cardiovascular dysautonomia in Achalasia Patients: Blood pressure and heart rate variability alterations
Source: PLoS One. 2021 Mar 15;16(3):e0248106. doi: 10.1371/journal.pone.0248106 (PMC7959365; doi:10.1371/journal.pone.0248106)
Supplement: S1 Fig — (PDF) [file pone.0248106.s001.pdf]

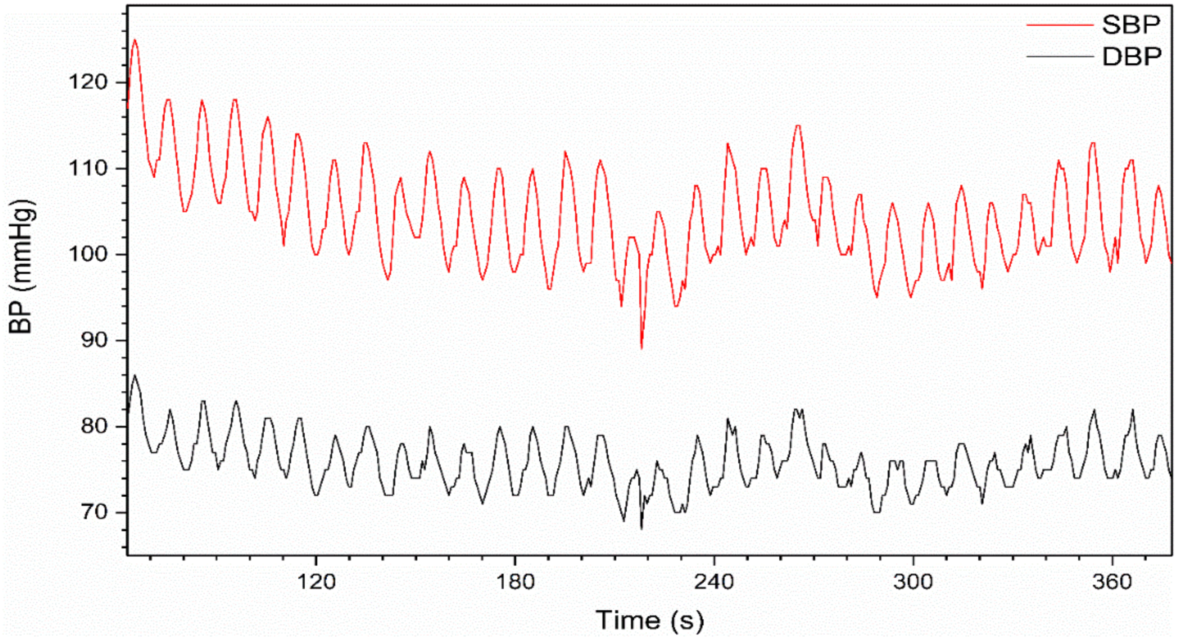

S1 Fig. SBP and DBP of a control man 21 years old breathing rhythmically at 0.1 recorded by the Portapress®.
