# Supplementary figures and images for: Cardiovascular dysautonomia in Achalasia Patients: Blood pressure and heart rate variability alterations
Source: PLoS One. 2021 Mar 15;16(3):e0248106. doi: 10.1371/journal.pone.0248106 (PMC7959365; doi:10.1371/journal.pone.0248106)

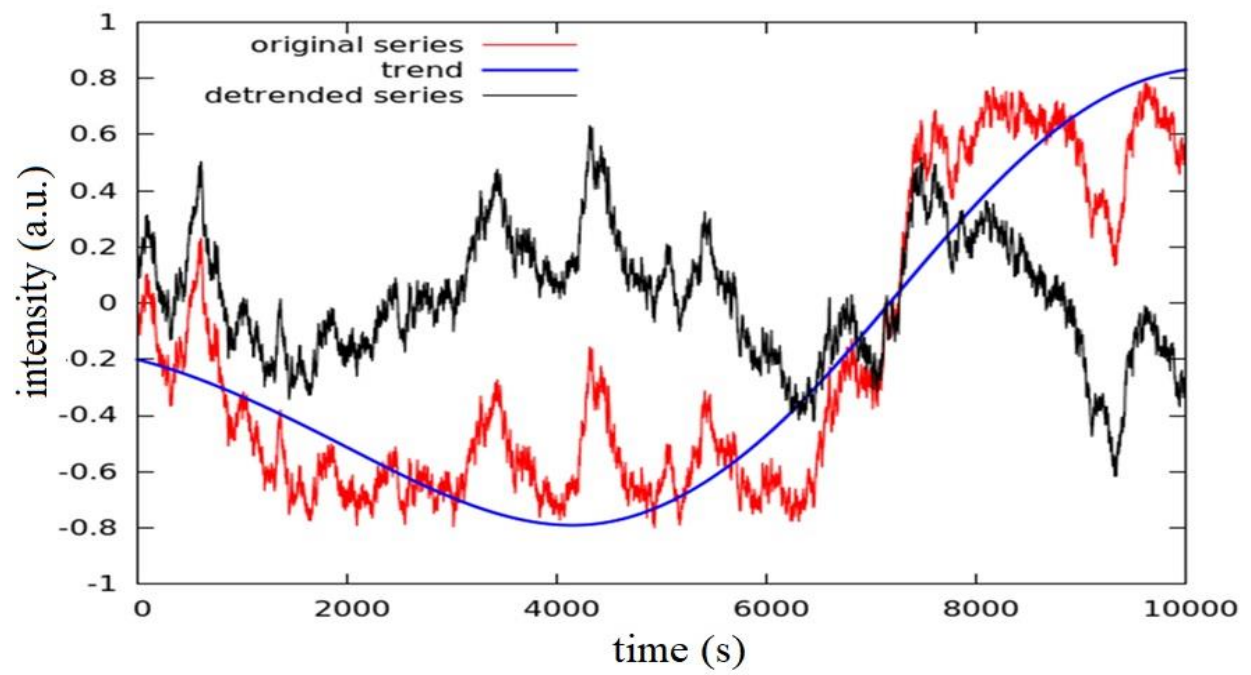

**S2 Fig.** Trend removal using Empirical Mode Decomposition.

Supplement: S2 Fig — (PDF) [file pone.0248106.s002.pdf]
