# Supplementary material for: Cardiovascular dysautonomia in Achalasia Patients: Blood pressure and heart rate variability alterations
Source: PLoS One. 2021 Mar 15;16(3):e0248106. doi: 10.1371/journal.pone.0248106 (PMC7959365; doi:10.1371/journal.pone.0248106)
Supplement: S3 Fig — Dots are detrended data points and continuous line is interpolation. (PDF) [file pone.0248106.s003.pdf]

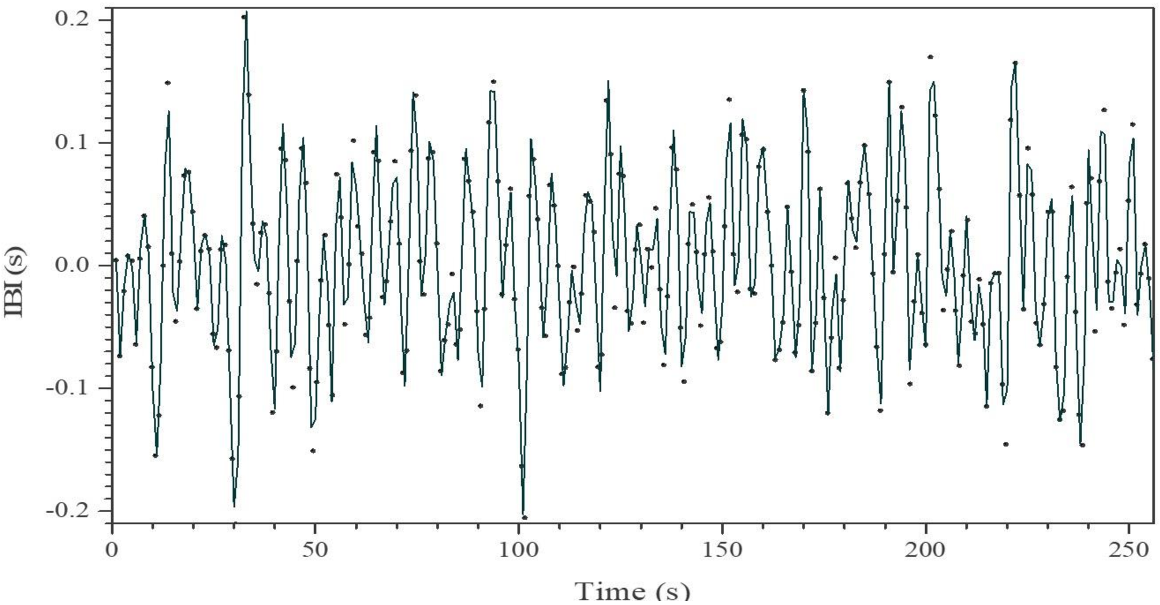

S3 Fig. Equidistant time-series of the interbeat interval (IBI) generated by cubic-spline interpolation. Dots are detrended data points and continuous line is interpolation.
