# Supplementary material for: Cardiovascular dysautonomia in Achalasia Patients: Blood pressure and heart rate variability alterations
Source: PLoS One. 2021 Mar 15;16(3):e0248106. doi: 10.1371/journal.pone.0248106 (PMC7959365; doi:10.1371/journal.pone.0248106)
Supplement: S1 Appendix — (DOCX) [file pone.0248106.s004.docx]

**Appendix: Analysis methodology**

All IBI records obtained with the Portapress^®^ were visually inspected for ectopic beats or abnormal intervals, and initial transient effects or artefacts were excluded from the data. To avoid contributions of artificial trends to the low-frequency regions of the spectrum, and to normalize the baseline to zero for all recordings, signals were detrended using the Empirical Mode Decomposition (EMD) technique with a program in C [56, 74], see S2 Fig. Subsequently, in OriginPro^®^ 2017, 64 bits, an equally-spaced time series (with a sampling rate of one second) was generated from the irregularly sampled data using a cubic spline interpolation (S3 Fig). The equally-spaced time series differed only slightly from the original signal, but interpolation was performed to ensure the applicability of spectral methods based on the Fourier transform (FT) [50, 55]. FT allow us to decompose the time series into its fundamental frequencies.

Here, we evaluated the statistical moments of IBI and SBP using OriginPro^®^ (2017, 64 bits) and independently checked using a code written in Python^®^ 2.7.7:

- Mean (*m*), a central moment measure. For IBI, *m* is inversely proportional to the heart rate.
- Standard deviation (SD), the second moment, gives the total power of spectral analysis, and a measure of the “rigidity” of the distribution. SD is one of the HRV measures of vagal and sympathetic modulation.
- Skewness (*sk*), the third moment, a measure of the symmetry of the distribution. IBI for control subjects have a marked asymmetry toward the right-hand side of the distribution [75]. It has been proposed that asymmetric tails to the left or to the right reflect, respectively, the acceleration or deceleration capacity of the heart rate as an approximate distinction of vagal and sympathetic effects on the cardiac modulations [46], thus *sk* reflects the balance of vagal and sympathetic effects.
- Kurtosis, (κ) the fourth moment, a measure of how much the data is concentrated around the mean reflecting not only the “rigidity” of the time series but also its “planarity”. For a Gaussian (normal distribution), the kurtosis is zero, while positive κ corresponds to a leptokurtic distribution (more peaked than a Gaussian) and negative κ describes a platykurtic one (flatter than a Gaussian).

In order to quantify simultaneously heart rate and blood pressure variability, we collected the various moments in a single measure through the HMP α defined as [54, 56]:

$$\alpha= \frac{\left[ \sqrt{\left( \frac{\mathrm{SD}}{m} \right)^{2}+\text{sk}^{2}+\kappa^{2}} \right]_{\text{IBI}}}{\left[ \sqrt{\left( \frac{\mathrm{SD}}{m} \right)^{2}+\text{sk}^{2}+\kappa^{2}} \right]_{\text{SBP}}} .$$

Spectral analysis of IBI records quantifies heart rate variability in different spectral bands: a low-frequency (LF) region from 0.04 to 0.15 Hz considered as a marker of sympathetic modulation, and a high-frequency (HF) region from 0.15 to 0.4 Hz associated with cardiac vagal activity [50, 51]. Even when most of the literature considers LF as a marker of sympathetic modulation [76], there are some that have doubts of this interpretation [77, 78]. To perform the spectral analysis, Fast Fourier Transform (FFT) was applied to short segments of 256 successive data points of the resulting detrended and interpolated record (equivalent to the last 4 minutes and 16 seconds of the record), then LF and HF were evaluated calculating the area under the FFT magnitude of the IBI signal from 0.04 to 0.15 Hz (LF) and from 0.15 to 0.40 Hz (HF). Units for LF and HF are s^2^. The LF/HF ratio has been used as a measure of the sympathetic/vagal balance; an increase of LF/HF is assumed to reflect a shift towards sympathetic dominance, whereas a decrease of this index has been interpreted as a parasympathetic dominance [50, 52]. We also evaluated the frequency radius (r_f_) ($\sqrt{\text{LF}^{2}+\text{HF}^{2}}$) [55] as an alternative way to measure the total modulation capacity of the autonomic nervous system, including contributions from both the sympathetic and the vagal branches. Moreover, by minimum least squares was used to find the slope of the Power Spectral Density (PSD, the logarithm of the FFT magnitude) as function of the logarithm of the frequency.

It is known that for control subjects, the respiratory resonant frequency of 0.1 Hz can induce periodic modulations in the cardiac rhythm [79]. This produces a narrow band around the breathing frequency evident in the Fourier transform of the IBI signal during a controlled breathing test. To quantify the strength of the cardiorespiratory coupling, we evaluated the respiratory peak (RP) as the power in the frequency band centered at the peak in the 0.1 Hz region spanning from the starting rising position to the end decreasing point (generally from 0.086 Hz to 0.113 Hz). If there was no a clear respiratory peak, the power in the region from 0.086 to 0.113 Hz was considered as the RP [56]. In order to quantify the relative importance of the cardio-respiratory coupling in a way that is not sensitive to the total power in the spectrum, we used the resonance parameter β $\left( \frac{\text{RP}}{\left( \text{LF}+\text{HF} \right)} \right)$ [55].
